# A new method allowing long‐term potentiation recordings in hippocampal organotypic slices

**DOI:** 10.1002/brb3.692

**Published:** 2017-04-12

**Authors:** Paula Paci, Sylvain Gabriele, Laurence Ris

**Affiliations:** ^1^ Neuroscience UnitResearch Institute for Health Sciences and TechnologyUniversity of Mons – UMONSMonsBelgium; ^2^ Mechanobiology & Soft Matter GroupInterfaces and Complex Fluids LaboratoryResearch Institute for BiosciencesUniversity of Mons – UMONSMonsBelgium

**Keywords:** adaptor, culture insert, electrophysiology, recording chamber, SKF

## Abstract

**Background:**

Hippocampal organotypic slices are used to improve the understanding of synaptic plasticity mechanisms because they allow longer term studies compared to acute slices. However, it is more delicate to keep cultures alive in the recording system outside *in vitro* conditions. Experiments from the organotypic cultures are common but the handling of slices is rarely described in the literature, even though tissue preservation is crucial. Instruments are sometimes required to extract the slices from the culture inserts but this approach is delicate and can lead to damage, given how strongly the slices are attached to the insert.

**Methods:**

A new configuration is proposed to secure the transfer of slices from the incubator to the recording chamber through an adaptor piece that can be designed for any model of chamber and/or insert. The adaptor is a Plexiglas ring in which a culture insert containing the slice can be easily introduced and stabilized. This system allows slices to be placed in the interface for electrophysiological investigations without having to detach them from the insert. That way, no damage is caused and the recording system can safely hold the slices, maintaining them close to culture conditions.

**Results:**

In addition to the description of the adaptation system, slices were characterized. Their viability was validated and microglial expression was observed. According to the experimental conditions, neuroprotective ramified microgliocytes are present. Dendritic spines studies were also performed to determine neuronal network maturity in culture. Moreover, SKF 83822 hydrobromide and three trains of 100 pulses at 100 Hz with a 10‐min inter‐train interval are suggested to induce long‐term potentiation and to record an increase of fEPSP amplitude and slope.

**Conclusion:**

This paper provides detailed information on the preparation and characterization of hippocampal organotypic slices, a new recording configuration more suitable for cultures, and a long‐term potentiation protocol combining SKF and trains.

## 1 Introduction

Long‐term potentiation (LTP) of synaptic transmission in the hippocampus constitutes the first experimental model for investigating the processes underlying learning and memory in vertebrates. The LTP mechanism is dependent on the activation of N‐methyl‐D‐aspartate (NMDA) receptors located in the postsynaptic membrane of neurons. These neurons trigger calcium ion entry leading to presynaptic and postsynaptic molecular pathways responsible for a persistent increase in synaptic efficacy (Bliss & Collingridge, 1993).

Mechanisms of synaptic plasticity have been abundantly studied from acute hippocampal slices, which have allowed the identification of the molecular processes of LTP induction and maintenance (Schwartzkroin & Wester, 1975). However, these investigations are limited by time because acute slices can be investigated from 6 to 12 hr, or at most 36 hr, using the system proposed by Buskila et al. (2014).

In order to carry out longer term studies, hippocampal cultured slices can be used. The culture of nervous tissue to study both normal and diseased brain functions is well‐known and very well established for a variety of brain regions. It consists of an *ex vivo* structure replicating many aspects of the *in vivo* context. This system presents a number of advantages over animal models, such as easy access and precise control of the extracellular environment, which is crucial in the study of molecular pathways underlying the synaptic plasticity (Cho, Wood, & Bowlby, 2007). For studies of the hippocampus, slices are taken from the neonate rats and present basic connections which become progressively elaborated to form a mature synaptic network. This network has appropriate regional differentiation imitating the endogenous developmental changes in the hippocampus during the first few weeks after birth (Bahr, 1995; Muller, Buchs, & Stoppini, 1993). The culture of slices can be achieved through different procedures, such as the roller tube technique (Gähwiler, 1981) or the interface method, which maintains slices on a porous membrane at the interface between the culture medium and humidified air by using culture inserts. As such, the oxygenation of slices is optimized while providing adequate nutrition by capillarity (Stoppini, Buchs, & Muller, 1991). The latter is a simpler method and allows the preservation of the organotypic organization of the tissue, contrary to the roller tube technique which results in a monolayer aspect of slices. The preservation of tissue architecture is relevant for studies of the physiological mechanisms of slices, moreover, it permits the interactions of multiple cell types, such as glial cells, which are crucial for neuron survival. As such, this model presents long‐term maintenance and can be utilized to evaluate the LTP and synaptic efficacy in different experimental conditions, allowing for a better understanding of the molecular mechanisms of memory formation and the facilitation of cognitive function.

Different protocols for electrophysiological investigations carried out on hippocampal organotypic slices are mentioned in the scientific literature but method chapters are often succinct, particularly when detailing recording chambers that must be adjusted to culture systems. Nevertheless, Rambani, Vukasinovic, Glezer, and Potter (2009) proposed a structured plan of a three‐dimensional microperfusion system to enhance the viability of thick brain slices associated with electrophysiological experiments but this system is technically complex and thus difficult to implement for experiments which require basic organotypic slices. Other teams have suggested a detailed representation of an interface chamber model but this system is specific to multisite recordings (Duport, Millerin, Muller, & Corrèges, 1999; Stoppini, Duport, & Corrèges, 1997).

This work was performed to provide detailed information on the preparation of hippocampal organotypic slices. The method used has been adapted from previously used methods (Muller, Toni, Buchs, Parisi, & Stoppini, 2001; Stoppini et al., 1991) and demonstrates that slices obtained in this way are viable, preserve the organization of hippocampal neurons and develop mature dendritic spines in culture. In particular, this paper recommends a new recording configuration ensuring cultured slice integrity to improve the achievement of LTP studies using this model. An adaptor piece has been designed for this purpose, and is easily reproducible using the provided technical plan. Finally, a long‐term potentiation induction protocol combining the SKF 83822 hydrobromide perfusion, a selective dopamine D_1_‐like receptor agonist and three trains of 100 pulses at 100 Hz with a 10‐min inter‐train interval is suggested. The choice of molecule was made based on the fact that dopaminergic fibers are important to long‐term potentiation in the hippocampus and because they degenerate in organotypic cultures contrary to acute slices.

## 2 Material and Methods

### 2.1 Hippocampal organotypic tissue cultures

All animal procedures were carried out according to European Community Council guidelines for the care and use of animals in research, and with the agreement of the local ethics committee.

Hippocampal organotypic slices were taken from 7 to 8‐day‐old Wistar rat pups of internal breeding (Neuroscience Unit, UMONS, Mons, Belgium) adjusting the original protocols (Muller et al., 2001; Stoppini et al., 1991). The points modified from the original protocols concern the glucose concentration in the culture medium, which was 5.6 mmol/L and 36 mmol/L, respectively, in Stoppini and Muller's protocols. It was adjusted to 11.2 mmol/L in this study to approach the glucose concentration in the aCSF. No instruments, such as spatulas, were used to manipulate the slices and lapped glass pipettes were preferred.

Dissection and culture preparation were performed under a laminar flow hood in sterile conditions. Instruments were sterilized for 2 hr at 180°C and the material was placed under germicide UV illumination for 30 min after 75% ethanol cleaning. The work surface was washed with 75% ethanol or Meliseptol^R^ (B. Braun Medical AG, Switzerland) and the laminar flow hood and incubators were regularly sprayed with Biocidal ZFTM (WAK‐Chemie Medical GmbH, Germany) according to firm recommendations.

The animals were quickly decapitated and their brains isolated in a Petri dish (82.1194 Sarstedt) with a cold sterile dissection medium. This solution consisted of Minimum Essential Medium Eagle with Earle's salts and L‐glutamine (M0268 Sigma), 25 mmol/L HEPES (HN77.3 Roth), 128 mmol/L NaCl (S7653 Sigma‐Aldrich), 5.6 mmol/L glucose (G5767 Sigma‐Aldrich), 10 mmol/L Tris (93362 Sigma) and 1% penicillin‐streptomycin (15140–148 Life Technologies). The hippocampi were dissected with binoculars and placed on the Teflon membrane of the McIlwain Tissue Chopper (The Mickle Laboratory Engineering Co. LTD, UK) so that the section was transversal to the longitudinal axis of the hippocampus. The liquid around the tissue was carefully eliminated before cutting slices of 400 μm thickness. In a large Petri dish (821473 Sarstedt), the slices were separated using up and down with a grinded glass pipette and each slice was controlled using a binocular microscope to choose only slices presenting both complete cornu Ammonis (CA) and dentate gyrus (DG) areas. The selected slices were put on culture inserts (PICM01250, PICM0RG50 Merck Millipore) and placed in cell culture multiwell plates (662160, 657160 Greiner Bio‐One International) which had been filled beforehand with a preheated culture medium (180 μl for small inserts, 1 ml for large inserts). The culture medium was composed of 50% Minimum Essential Medium Eagle with Earle's salts and L‐glutamine (M0268 Sigma), 25% Horse serum (26050–088 Life Technologies), 25% Hank's Stock Solutions with CaCl_2_ and MgCl_2_ (14025–050 Life Technologies), 1% penicillin‐streptomycin (15140–148 Life Technologies), 5 mmol/L Tris (93362 Sigma), 0.03% NaHCO_3_ (S8875 Sigma‐Aldrich) and 11.2 mmol/L glucose (G5767 Sigma‐Aldrich) at pH 7.2. Small culture inserts (PICM01250 Merck Millipore) were used for the electrophysiology tests while large culture inserts (PICM0RG50 Merck Millipore) were selected for immunohistochemistry and biolistic transfection. Three confetti of 7 mm diameter, handmade from sterilized hydrophilic polytetrafluoroethylene (PTFE) membrane (FHLC04700 Merck Millipore), had been placed in large culture insert beforehand to facilitate slice manipulations for microscope observations. Confetti were used in order to facilitate the subsequent recovery of the slices using fine pliers without having to detach them from the insert membrane. The slices were maintained in a humidified atmosphere at 37°C with 5% CO_2 _for 4 days. Then, they were transferred to another incubator at 33°C. The culture medium was replaced every 3 days under a laminar flow hood with preheated fresh medium.

### 2.2 Viability in culture

The selected slices were observed from culture plates with an optical microscope (Carl Zeiss Axiovert 25C, Germany) at X50 magnification and images were acquired with a digital camera (Deltapix DP 200, Denmark) from 0 to 24 days *in vitro*.

Twelve slices from two different cultures were incubated with propidium iodide (PI) (P4864 Sigma‐Aldrich) diluted at 5 μg/ml in culture medium. This solution was replaced every 3 days in culture. The slices incubated with PI were investigated from the culture plates with a fluorescence inverted microscope (Olympus IX70, Japan) at X20 magnification to visualize whole surface of the slice, the cornu Ammonis or the gyrus dentate specifically defining regions of interest (ROI). The excitation light wavelength was 535 nm and a rhodamine filter (>590 nm) was applied with an exposure time of 1000 ms for all observations. Fluorescent digital images were taken with a digital camera (IMAGO–TILL PHOTONICS, Germany) and image processing was performed with TILLvisION 4.0 analysis software (TILL PHOTONICS, Germany). The average of fluorescence intensity was measured from the ROI of each slice after 2, 6, and 10 days of culture, and values were compared by one‐way repeated measures of analysis of variance. Positive controls were carried out putting one 10‐day slice at 4°C for 2.5 days.

### 2.3 Microglial expression

Twenty‐two‐day slices were rinsed 3 times briefly in phosphate‐buffered saline (PBS) at pH 7.4 then fixed overnight at 4°C in 4% paraformaldehyde (P6148 Sigma‐Aldrich) with 0.1% Triton X‐100 (X100 Sigma). After three washings of 15 min in PBS, the slices were incubated in 0.1 mol/L glycine solution (220910010 Acros Organics) for 2 hr. The permeabilization of the slices was performed in two steps, first using 0.3% Triton X‐100 solution for 1.5 hr, and then using hyaluronidase solution (H2126 Sigma‐Aldrich) at 1500 units/ml for 45 min. Non‐specific sites were blocked in 2% BSA (134731000 Acros Organics) with 0.3% Triton X‐100 solution for 2 hr before incubating the slices overnight at 4°C in 50 μl of an anti‐Ionized calcium‐binding adaptor molecule 1 (Iba1) primary antibody (019–19741 Wako Pure Chemical Industries, Ltd.) diluted in blockage solution (1/100). Three washings in PBS were carried out for 30 min and a second blockage step was performed. The slices were incubated for 2 hr and protected from light in a drop of 50 μl of an anti‐IgG rabbit IgG (H + L) polyclonal secondary antibody (A‐11008 Invitrogen) diluted in PBS with 0.25% Tween 20 (P1379 Sigma‐Aldrich) (1/100). After three washings in PBS and three washings in distilled water, the slices were mounted in a drop of Vectashield with DAPI (H‐1200 Vector Laboratories, Inc). The staining was observed at ×20 magnification with an Olympus FV1000 Inverted Confocal IX81 Microscope and confocal images were acquired in the cornu Ammonis with FV10‐ASW 4.1 software (Olympus).

### 2.4 Dendritic spine detection

Twenty‐day‐old slices were incubated with phalloidin (R415 Invitrogen 1/50) in the same way as previously mentioned, but without the secondary antibody step given that phalloidin was paired to rhodamine. Co‐staining with an anti‐microtubule‐associated protein 2 (MAP2) primary antibody (AB5622 Millipore 1/50) was carried out by simply blending both primary antibodies, and an anti‐IgG rabbit IgG (H + L) polyclonal secondary antibody (A‐11012 Invitrogen) was used against the anti‐MAP2 antibody. Confocal images were acquired from the cornu Ammonis at 60× magnification using an Olympus FV1000 Inverted Confocal IX81 Microscope and FV10‐ASW 4.1 software (Olympus).

Seven‐day‐old slices were transfected using a biolistic method (Helios Gene Gun System, BIO‐RAD Laboratories Ltd., United Kingdom) according to BIORAD's instructions. The tubing was cleaned with 100% ethanol (32221 Sigma‐Aldrich) and completely dried in the tubing prep station by purging nitrogen (0.3–0.4 L/min) for at least 10 min. In a 1.5 ml microtube, 5 mg of gold particles of 1.6 μm diameter (1652264 BIO‐RAD) were added to 50 μl of 50 mmol/L spermidine (S2626 Sigma) diluted from the stock solution in 100% ethanol. This mixture was vortexed briefly and sonicated three times for 10 s. Then, 5 μg of deoxyribonucleic acid (DNA) (pEGFP‐N2 6081‐1 Vector Clontech), generously supplied by the Molecular Biology Unit (UMONS), were added and very slightly mixed. Twenty‐five μl of 2 mol/L CaCl_2 _(CN93.1 ROTH) were added to allow DNA precipitation for 10 min. The enhanced green fluorescent protein (EGFP) vector had been amplified and purified beforehand, according to the instructions of the NucleobondXtra Maxi EF (740426.10 Macherey‐Nagel) from C600 bacteria (Molecular Biology Unit, UMONS). The supernatant was removed and discarded and three washings with 500 μl of 100% ethanol were performed. Between the washings, the tube was spun at 1000 rpm for a few seconds in a microcentrifuge. The pellet was suspended in 200 μl of polyvinylpyrrolidone (PVP) solution (Sigma PVP360) at 0.05 mg/ml and the mixture was transferred into a 15 ml Falcon tube. The remains of the gold particles were recovered with PVP solution to obtain a 1.5 ml final volume. The gold suspension was loaded into the tubing, which had been cleaned and dried with an adapted syringe (300613 BD Plastipak^™^) to avoid bubbles forming inside the tubing. This was turned for 2 min in the prep station before the microcarriers settled for 30 s. The ethanol was removed very slowly from the tubing with a syringe, and the tubing containing the coated particles was dried with nitrogen flow for around 15 min at 0.4–0.5 L/min. It was then cut into cartridges which were used directly, or stocked for 2–3 months in a desiccated environment at 4°C for later experiments. Storage condition recommendations were respected, especially the use‐by dates for spermidine and PVP. The slices were transfected at 7 days *in vitro* at 120 psi with an additional spacer of 1 cm from slices using a nylon mesh. After 11 days (DIV18), the transfected slices were fixed in 4% paraformaldehyde (P6148 Sigma‐Aldrich) with 0.1% Triton X‐100 (X100 Sigma) overnight at 4°C.

### 2.5 Quantitative and qualitative dendritic spine analysis

Six pyramidal neurons were randomly selected from the transfected slice cultures and a three‐dimensional confocal reconstruction was performed. This was performed with an Olympus FV1000 Inverted Confocal IX81 microscope using a 60× oil‐immersion objective (UPLSAPO), with a numerical aperture of 1.35, connected to FV10‐ASW 4.1 software (Olympus), as a Z‐series of 14–80 images averaged 3 times (Line Kalman) with 0.41 μm intervals, 1024 × 1024 pixel resolution at a scan speed of 40 μs/pixel. Several pictures were acquired to reconstitute entire neurons and the images were modeled semi‐automatically using the Filament Tracer Imaris software (Bitplane, UK). Some parameters were measured on dendrites (length and number of spines) and on dendritic spines (length, largest diameter of spine head and finer diameter of spine neck) from a series of pictures assembling the whole area of apical dendrites for each investigated neuron. Spine density was calculated by dividing the total spine number (corresponding to the sum of spines obtained from different pictures) by the total dendrite length (corresponding to the sum of dendrite lengths obtained from different pictures) and the dendritic spines were classified as stubby‐, mushroom‐ and thin‐shaped spines. In order to sort them, the spine lengths and ratios between the maximum spine head and the minimum neck diameter were used according to the following criteria: stubby (length < 1 μm, max_head_/min_neck_ratio < 1.5), mushroom (max_head_/min_neck_ ratio ≥ 1.5, independent of spine length), thin (length ≥ 1 μm, max_head_/min_neck_ ratio < 1.5) (Weyer et al., 2014; Zagrebelsky et al., 2005). The values obtained were exported into Microsoft Excel and were converted into a matrix to perform an automatic classification with R software. Boxplots were created to represent the percentage of each spine class.

### 2.6 Cross‐section validation of hippocampal organotypic slices

Immunohistochemistry was performed on 25‐day slices, in accordance with the protocol detailed previously, with a multi‐species primary antibody blend (Milli‐Mark^™^ChromaPan Neuronal Marker‐OMC NS330 Millipore) (1/50) and a single multi‐species secondary antibody blend (NS330 Millipore) containing different fluorophores (1/100). The staining was observed at ×10 magnification using an Olympus FV1000 Inverted Confocal IX81 Microscope, and confocal images were acquired in the cornu Ammonis with FV10‐ASW 4.1 software (Olympus).

### 2.7 New recording configuration

**Figure 1:**
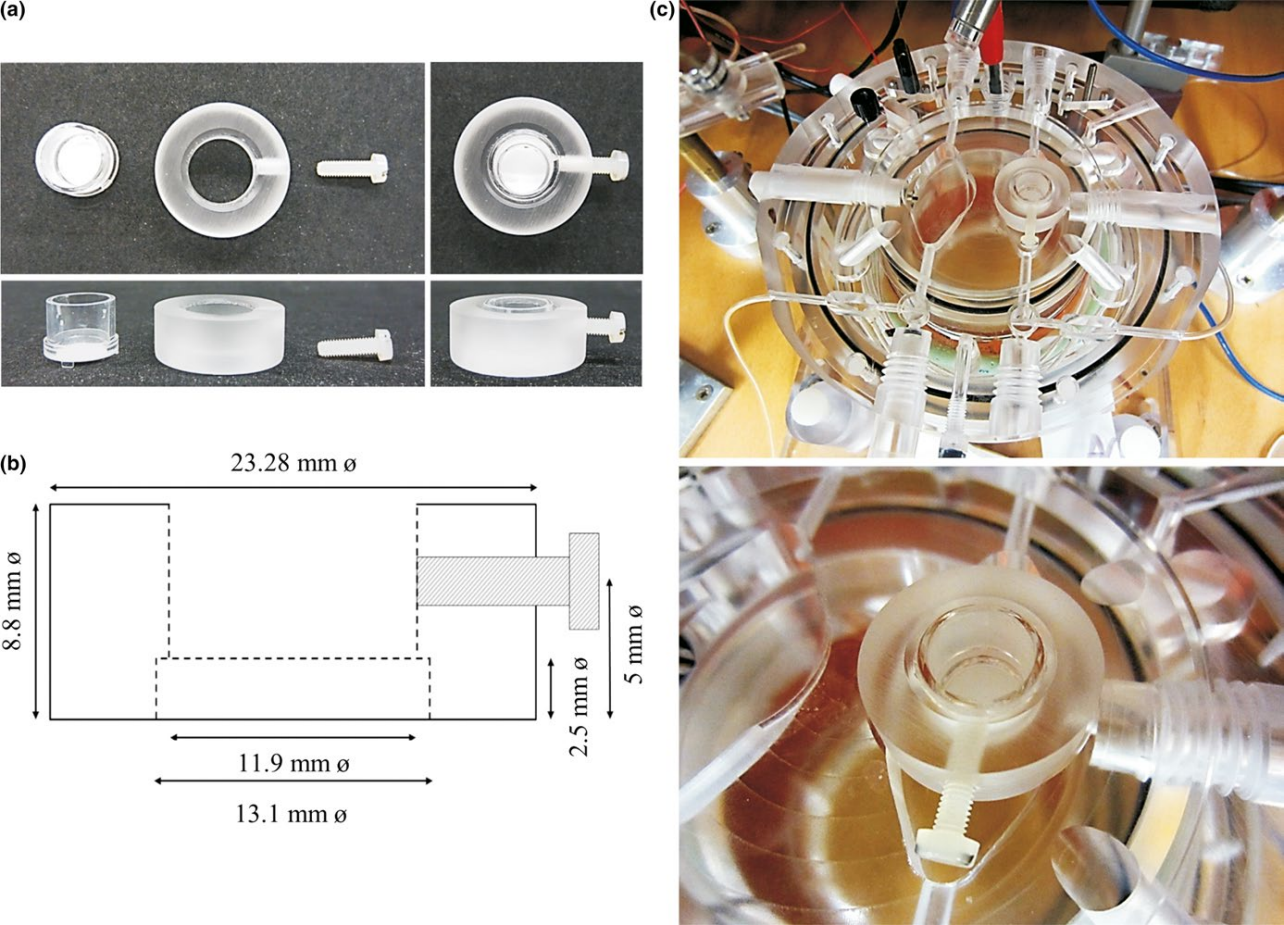
Adaptation of the recording chamber to hippocampal organotypic slices. (a) An adaptor unit was designed in function of the respective dimensions of culture inserts and recording chamber sizes. To secure the insert in the adaptor, a nylon screw was required. From left to right, pictures show firstly the culture insert, the adaptor and the screw separately, and secondly the three parts assembled. The above and side views are represented. (b) Schematic plan of adaptation system. The culture insert is represented by a dotted line, the adaptor by a full line and the screw is in gray. (c) System positioning in the recording chamber. Pictures display the interface recording chamber holding the adaptation system (above) with an enlargement (below)

To secure the handling of hippocampal organotypic slices for electrophysiological investigations while maintaining conditions close to those encountered in culture, the recording chamber was adapted to the culture system using an adaptor unit. The adaptor unit consists of a Plexiglas ring, designed in function of the respective dimensions of culture insert and chamber sizes, in which the insert containing the slice can be placed. To secure the insert in the adaptor, a nylon screw is required. Figure 1a shows the culture insert, the adaptor, the screw and the way they interlock (from left to right). Figure 1b precisely illustrates the system dimensions with culture insert represented by a dotted line, the adaptor by a full line, and the screw in gray. Figure 1c represents a picture and an enlargement of the proposed system placed inside the recording chamber. This new recording configuration was adapted to an interface recording chamber (Tissue Slice Recording Chamber dual well 21051‐00 Fine Science Tools, Canada) but can be adjusted to different chambers and culture insert models.

### 2.8 Electrophysiology

A protocol proposed by Villers and Ris (2013) was used. All the electrophysiological experiments were reproduced in the same environment with identical equipment and all the slices, from day 7 to day 44 *in vitro*, were treated in the same way in an interface recording chamber (Tissue Slice Recording Chamber dual well 21051‐00 Fine Science Tools, Canada) (Figure 1c). A short rinse with artificial cerebrospinal fluid (aCSF; 124 mmol/L NaCl, 26 mmol/L NaHCO_3_, 10 mmol/L D‐glucose, 4.4 mmol/L KCl, 1.3 mmol/L MgSO_4_, 1 mmol/L NaH_2_PO_4_, 2.5 mmol/L CaCl_2_ pH 7.4) preheated at 33°C was used to eliminate the culture medium. It simply consisted of placing the culture insert (PICM01250 Merck Millipore) containing the slice into a large drop of aCSF inside a Petri dish. After washing, the insert was put into an adaptor piece, which in turn was placed into the recording chamber as illustrated in Figure 1. The hippocampal organotypic slices were not immersed but were kept in the interface for the electrophysiological investigations, adjusting the aCSF level to reach the base of the insert and avoiding bubbles between the insert‐adaptor complex and the bottom of the chamber. The slices were perfused at 2 ml/min using a peristaltic pump system (Minipuls 3 Gilson, USA) with aCSF for a few minutes before microelectrode positioning. The aCSF was preheated at 33°C using a bain‐marie and a temperature controller (TR‐200 FST, Canada), and oxygenated with a gaseous blend composed of 95% oxygen and 5% CO_2_. Stimulating electrodes were bipolar microelectrodes formed of two nickel‐chrome braided wires of 51 μm in diameter (762000 A‐M Systems, Inc., USA). Recording electrodes consisted of glass capillaries with a 1.5 mm diameter (TW150‐4 World Revision Instruments Inc., USA) stretched using a micropipette puller (P‐97 Sutter instrument, USA) and filled with aCSF to obtain a tip resistance of 2–5 MΩ. The electrodes were orientated precisely with Narishige micromanipulators and placed in the first area of the cornu Ammonis (CA1) (75–150 μm under the surface of the slice) at the *stratum radiatum* level. A bundle of Schaffer collaterals was stimulated to record field excitatory post‐synaptic potentials (fEPSPs) (Figure  7a). When the electrodes were lowered onto the slice, filter papers were carefully placed all around to close the recording chamber, stabilizing the temperature inside and preventing oxygen diffusion. Biphasic stimulation (0.08 ms pulse duration per half‐wave with 0.02 ms interval) was delivered 1/60s and applied at a constant voltage with a Grass stimulator (Grass Tech S88X Stimulator, Astro‐Med Inc., USA) connected to SIU‐V isolation units (Grass Tech, Astro‐Med Inc.). The maximal response was checked by increasing the stimulus intensity from 2 V to maximum 10 V. Field EPSPs were amplified 1000 times with a WPI ISO‐80 amplifier and filtered at 10 Hz and 10 kHz. The signal was then sent to a PC through a National Instrument A/D converter and the stimulation, data acquisition and analysis were performed using the WinLTP program (Anderson and Collingridge 2007) (www.winltp.com). The field EPSPs were adjusted to around 40% of the maximum amplitude obtained in an input‐output curve. The LTP was triggered by applying three different protocols. The first protocol consisted of applying three trains of 100 pulses (1 s) at 100 Hz separated by 10 min‐intervals. In the second protocol, the same procedure was applied but preceded by an incubation of 50 μmol/L forskolin (FSK) (Alomone Labs F‐500) for 5 min and in the third protocol, the trains were preceded by a 15 min‐incubation of 10 μmol/L SKF 83822 hydrobromide (SML0513 Sigma‐Aldrich), which is a selective dopamine D_1_‐like receptor agonist. The slices were classified into four groups (no basal response [NR] *n *= 35; not exploitable [NE] *n *= 13; exploitable *n *= 42 [no LTP *n *= 12 or LTP *n *= 30]). Slices without electrical activity (NR) or without a stable baseline (NE) were not considered further. According to the response profiles, different parameters were measured (amplitude, slope or spike amplitude). The amplitude was determined as the maximum amplitude of the response, the slope was measured around 30–60% of the peak amplitude, and for some slices showing population spikes, the spike amplitude was used to evaluate the efficacy of their synaptic transmission. Slices presenting the LTP were then classed into different groups depending on the parameter showing a potentiation (such as amplitude, slope, and spike LTP). For all the experiments under the SKF protocol (DIV10 to DIV22), the baseline was recorded in CA1 for 24 min and the LTP was assessed by analyzing the mean slopes or amplitudes of the fEPSP measured every minute for 84 min after the stimulation trains (every point was averaged from four successive measures). A relative percentage of potentiation after induction was calculated in function of the averaged values obtained from the baseline. In the results section, data will be presented by the mean ± SEM. The proportions of slices presenting both amplitude and slope LTP were calculated and compared between the three groups.

## 3 Results

### 3.1 Viability of hippocampal organotypic tissue cultures

**Figure 2:**
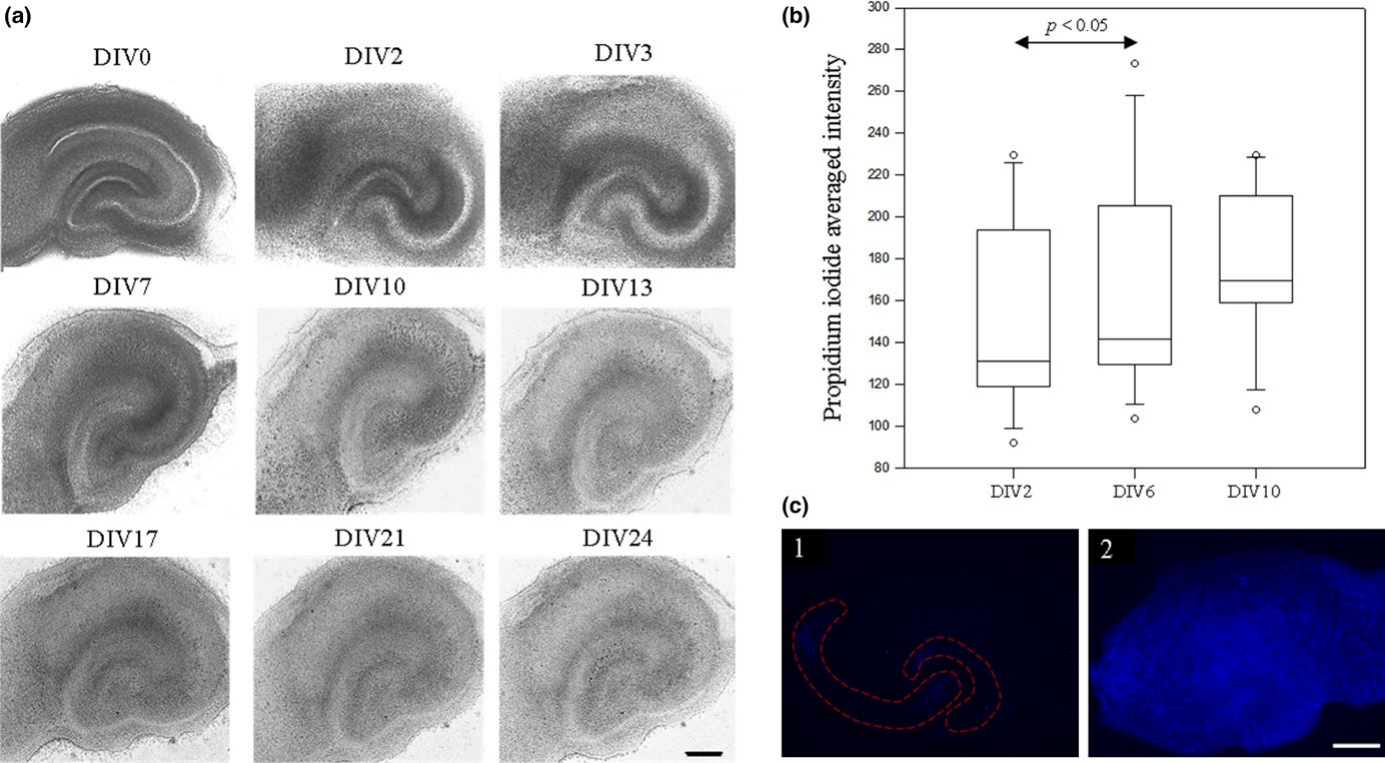
Hippocampal organotypic slices in culture. (a) Evolution of slices followed in the culture plate with light microscope at ×5 magnification. Every picture represents a slice from day 0 to 24 *in vitro*. (b) Quantification of cell death using propidium iodide (PI) fluorescence intensity. Slices were incubated with a PI solution diluted in the culture medium and observed after 2, 6 and 10 days. Fluorescence of the whole area of slices was visualized with a fluorescence microscope connected to a camera (magnification ×20) and measured with TILLvisION software. Values were plotted for every group where a significant increase was only detected between 2 and 6 days (*p* < .05). (c) Fluorescence acquisition of a slice incubated with PI. The first picture shows a slice incubated for 10 days (1) and the second picture represents the maximum fluorescence in a dead slice (after 2.5 days at 4°C) (2). Fluorescence microscopy was used at ×2 magnification and regions of interest (ROI) were defined in this way with TILLvisION software for each picture (dotted lines). Scale bars are 1 mm

Before any investigations, the viability of the hippocampal organotypic slices was tested. The first experiment consisted of following the evolution of the slices with light microscopy to evaluate necrosis, represented by dark areas and related to the degeneration of fibers and cells which had been sectioned or damaged during dissection. The slices were photographed from day 0 *in vitro *(DIV0) to day 24 *in vitro* (DIV24) and the pictures showed that the slices brightened gradually over time, highlighting that necrosed tissues are progressively repaired by glia. Moreover, the organization of pyramidal neurons was preserved even in the long term according to the *in vitro* conditions. However, a culture flattening, while remaining three‐dimensional (Figure 2a), was observed, as has already been described for hippocampal organotypic slices (Buchs, Stoppini, & Muller, 1993).

The second experiment studied the cell death rate of slices in culture with propidium iodide (PI). This marker is a membrane non‐permeant fluorescent molecule binding nucleic acids allowing the staining of only dead cells, given that they have lost their membrane integrity. The cultures were visualized by epifluorescence microscopy and the averaged fluorescence intensity produced by the total slice area was measured semi‐automatically with TILLvisION software after 2, 6 and 10 days of culture (DIV2, DIV6 and DIV10). The results revealed a basal cell death level at the beginning of the experiment (DIV2) that increased significantly after 6 days (*p* < .05), but no more after 10 days (DIV10), according to the one‐way repeated measures analysis of variance. Statistical analysis was carried out for 12 slices, for which the values for DIV2, DIV6, and DIV10 were plotted (Figure 2b). Analysis was also performed for the cornu Ammonis and dentate gyrus separately, after manual delimitation, but no significant difference in fluorescence intensity was detected between the groups (*p* = .125 and *p* = .097 respectively with Friedman repeated measures analysis of variance on ranks).

A slice was chosen at random within a culture and incubated for 2.5 days at 4°C to compare the rate of cell death in culture to that which can be caused in the tested conditions. Pictures presented some small fluorescent areas in the cornu Ammonis and the dentate gyrus in culture (Figure 2c‐1) compared with a higher and homogeneous signal after incubation at 4°C (Figure 2c‐2).

### 3.2 Microglial activation in culture

**Figure 3:**
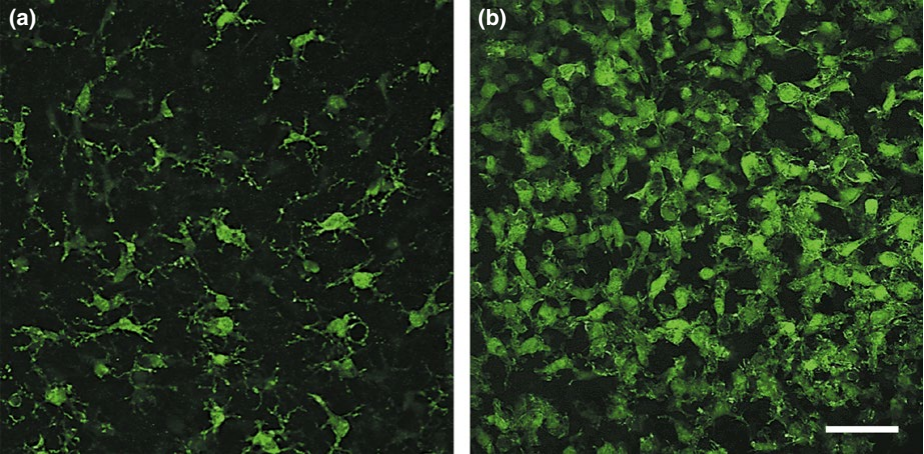
Activated microglia detection in hippocampal organotypic slices. Twenty‐two‐day slices were stained with an anti‐Iba1 antibody and fluorescent images were taken with confocal microscopy in the CA1 region at ×20 magnification in culture (a) and after lipopolysaccharides incubation (b). Scale bar is 50 μm

Microglial activation was also investigated to determine the basal inflammatory status of the slices in culture. Activated microglia was highlighted in 22‐day slices by immunohistochemistry using an anti‐Iba1 antibody. These cells were observed by the confocal microscopy to precisely see their morphology. Images from the CA1 region showed that a stellate phenotype, characterized by numerous fine branching processes, was detected in culture (Figure 3a) contrary to a macrophagic phenotype, which was specific to slices incubated in the presence of lipopolysaccharides (LPS). LPS, located in the outer membrane of bacteria, is known to elicit strong immune responses in animals mediated by microglia activation. Furthermore, in this condition, the density of the microgliocytes appeared higher and morphological change was observed with LPS, demonstrating that cultured slices present resting microglial cells (Figure 3b).

### 3.3 Maturation of hippocampal organotypic tissue cultures

In order to record synaptic activity and plasticity in cultured slices the neuronal connections must be mature. In order to verify the synaptic maturity of slices, the density and morphology of dendritic spines were examined by confocal microscopy. Two different methods were used, immunohistochemistry, and EGFP biolistic transfection.

**Figure 4:**
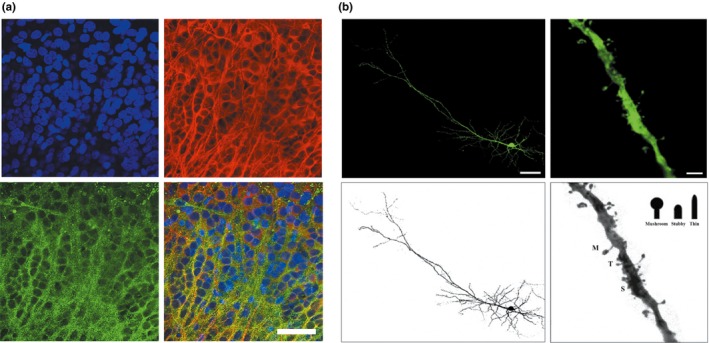
Dendritic spines in hippocampal organotypic slices. (a) Spine detection with phalloidin and anti‐MAP2 antibody in 20‐day slices, fluorescent images were obtained in the CA1 area with confocal microscopy at ×60 magnification. DAPI (blue), MAP2 (red), phalloidin (green), merge (right). Scale bar is 50 μm. (b) Spine display with enhanced green fluorescent protein (EGFP) biolistic transfection. Slices were transfected at DIV7 and 11 days after, pyramidal neurons of cornu Ammonis or dentate gyrus were observed with confocal microscopy at ×60 magnification. A 3D reconstruction was performed with FV10‐ASW 4.1 software by assembling multiple images of a single neuron (left). Numeric zoom (×10) was applied to confocal acquisitions to highlight dendritic spine morphology (right). Scale bars are 50 μm and 5 μm respectively

Firstly, 20‐day slices were incubated with rhodamine‐phalloidin, a soluble phallotoxin isolated from the deadly *Amanita phalloides* mushroom. This molecule targets F‐actin polymers that form dendritic spine cytoskeletons. Fluorescent microscopy revealed a dense punctuated labeling, suggesting numerous dendritic spines at the surface of cultured pyramidal neurons. A co‐staining with MAP2 was carried out for verification and pictures from the CA1 region showed a superimposition of these small fluorescent points along dendrites (Figure 4a).

**Figure 5:**
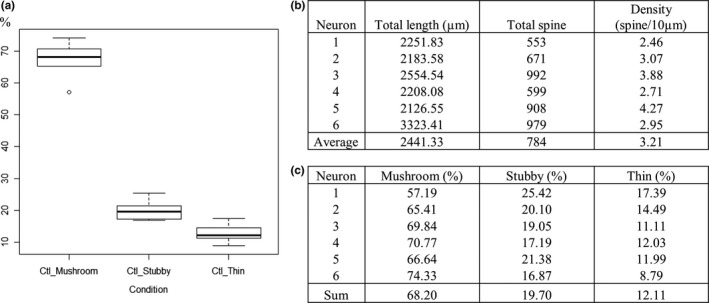
Quantitative and qualitative dendritic spine analysis. Six enhanced green fluorescent protein (EGFP)‐transfected pyramidal neurons were randomly selected and 3D confocal reconstruction was performed. A series of pictures assembling the whole area of apical dendrites for each investigated neuron were modeled semi‐automatically using the Filament Tracer Imaris software (Bitplane United Kingdom) and some parameters were measured on dendrites (length and number of spines) and on dendritic spines (length, largest diameter and finer diameter of spine neck). Values were used for spine classification and for spine density calculations. (a and c) Distribution of spine categories into mushroom‐, stubby‐ and thin‐shaped spines according to their length and the ratio between the maximum spine head and the minimum neck diameter. (b) Respective spine densities calculated from the total dendritic length and the total spine number for each neuron

Secondly, biolistic transfection was used. This technique presents the advantage of better identifying dendritic spines, given that labeling neurons are isolated and that any potential background noise is prevented. This method allows an EGFP‐expression vector to be introduced inside cultured slices using a low pressure helium pulse which delivers gold particles coated to DNA. The slices were transfected at DIV7 and the dendritic spines were investigated 11 days later at the surface of the transfected neurons after slice fixation. The three‐dimensional confocal reconstruction of 3‐week neurons from cornu Ammonis and dentate gyrus displayed numerous dendritic spines with thin, stubby or mushroom morphology (Figure 4b), as represented in Sorra's and Harris's work (Sorra & Harris, 2000). Here, the semi‐automatic analysis performed with the Filament Tracer Imaris software showed that in average of 3.21 spines were present per 10 μm of dendrite (Figure 5b), and a spine morphological study carried out on all neurons (with 4702 spines for *n *= 6) revealed that the most numerous spines were the mushroom‐type (68%), followed by the stubby (20%) and thin‐types (12%) respectively (Figure 5c). The proportion of each spine class from the separated neurons was plotted and the same distribution profile was observed (Figure 5a).

### 3.4 Cross‐section validation of hippocampal organotypic slices

**Figure 6:**
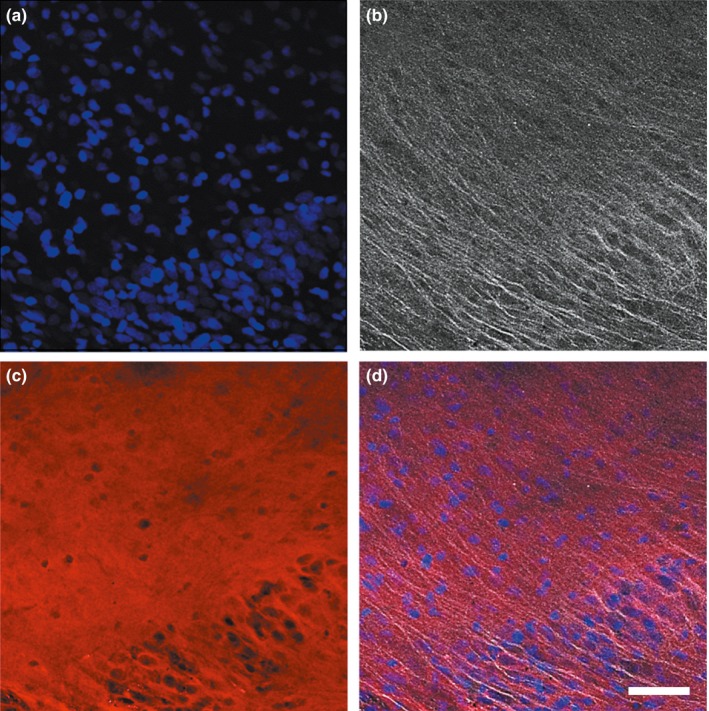
Axon and dendrite location in hippocampal organotypic slices. A blend of antibodies was used to simultaneously detect somatic, axonal, and dendritic proteins in 25‐day slices. Pictures were acquired with confocal microscopy at ×10 magnification. DAPI (a), soma‐dendrites (b), soma‐axons (c), merge (d). Scale bar is 50 μm

Prior to the electrophysiological studies it was verified that the axons and dendrites were located in the same plan, according to a cross‐section technique to allow appropriate stimulation and recording of cultured slices. A blend of primary antibodies targeting key axonal, dendritic, and somatic proteins, distributed across the neuronal architecture, was used to detect them simultaneously. Confocal images showed that the axons and dendrites were effectively co‐located in the 25‐day slices (Figure 6a–d).

### 3.5 Long‐term potentiation in hippocampal organotypic tissue cultures

**Figure 7:**
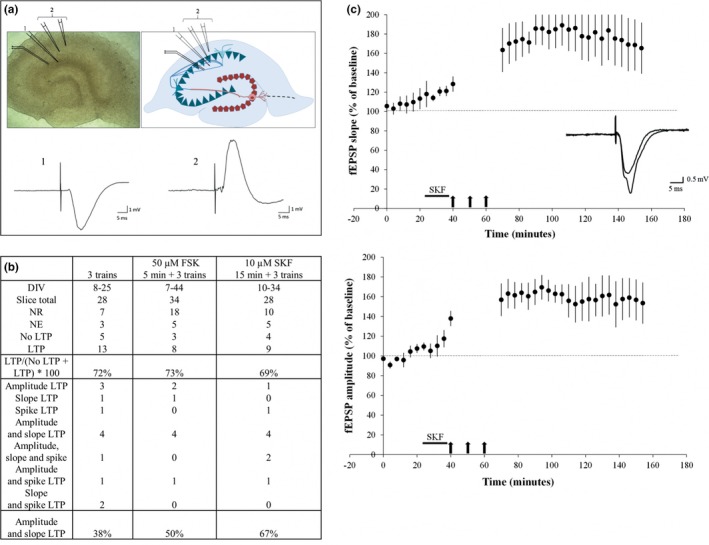
Long‐term potentiation (LTP) induction in hippocampal organotypic slices. (a) Positioning of stimulation and recording electrodes in CA1 and respective responses in an 11‐day slice. Picture (left) and schematic representation (right) are represented with the cornu Ammonis in blue and the dentate gyrus in red. Schaffer collaterals are shown using blue lines and mossy fibers using red lines. The stimulating microelectrode is placed in the CA1 region at the level of the Schaffer collaterals and the recording microelectrode in the apical dendrites of the *stratum radiatum *(1) or in the cell layer (2).* *Negative or positive response profiles are shown according to microelectrode positions. (b) Comparison of tested LTP protocols and related LTP profiles. Three different protocols were evaluated; three trains of 100 pulses at 100 Hz, 50 μmol/L forskolin (FSK) for 5 min followed by three trains and 10 μmol/L SKF for 15 min followed by three trains in a standardized way. Slices without response (No response [NR]) or without stable response (not exploitable (NE)) were removed prior to the assessment of the LTP percentages for each group. Different LTP categories were also detailed and the percentage of amplitude and slope LTP was calculated. (c) Field excitatory postsynaptic potentials (fEPSPs) recorded from 10 to 22‐day slices stimulated with SKF and trains protocol. A baseline was performed for 24 min, then the SKF perfusion (10 μmol/L for 15 min) was applied followed by three high frequency stimulation trains with 10‐min intervals (100 pulses 100 Hz) (black arrows). An immediate post‐tetanic potentiation is seen with an increase in the magnitude of the fEPSP both in amplitude (downwards) and in slope (upwards) for 84 min. Every point was averaged from four successive responses and error bars were represented (SEM with *n *= 6)

The first method consisted of electrical induction by the application of three stimulation trains of 100 pulses at 100 Hz (1 s), separated by 10 min‐intervals. The second method was a perfusion of 50 μmol/L FSK added to aCSF for 5 min, followed by the application of three stimulation trains, as previously. The last method was a perfusion of 10 μmol/L SKF added to aCSF for 15 min followed by the stimulation trains. The result revealed that the three protocols induced the LTP in the same way, with 72, 73, and 69% of slices showing an increase of their synaptic strength respectively in the CA1 region after induction. However, the third protocol (SKF 10 μmol/L 15 min + 3 trains) resulted in the largest proportion of both fEPSP amplitude and slope increase (Figure 7b). On average, amplitude and slope after induction represented 169 ± 10% and 176 ± 19% of the baseline, respectively, (Figure 7c) and could last between 1/2 hr to 4 hr according to the investigated organotypic slices, with 136 min on average.

## 4 Discussion

A simple and effective method for achieving hippocampal organotypic slices, adapted from Stoppini et al. (1991) and Muller et al. (2001) was used in this study. At the beginning the tissues presented necrosis, commonly caused by the dissection procedures. Nevertheless, this disappeared rapidly after a few days in culture as shown in Figure 2a. This evolution can be explained by an efficient cleaning of the damaged tissues achieved by glial cells that are essential for neuron survival.

Pictures also show that the architecture of the hippocampus was preserved even after 3 weeks in culture (Figure 2a), demonstrating that the interface culture method is optimal for slice maintenance, contrary to the roller tube technique, initially introduced by Gähwiler (1981), which can induce disorganization causing a monolayer aspect of tissues and the formation of holes.

To investigate the slice viability in culture, cell death was studied by fluorescence microscopy using a propidium iodide staining protocol. A signal was detected and measured on a whole area of slices after 2, 6, and 10 days in culture and the statistics show a significant increase after 6 days which did not intensify any more thereafter. In other words, the cell death rate increases during the first days in culture and then stabilizes (Figure 2b). Here, analysis of cell death intensity has concentrated on the cornu Ammonis and the dentate gyrus specifically. Results showed no significant difference between the groups demonstrating that slices present a basal neuronal death level which does not progress according to *in vitro* conditions. To determine if this cell death rate could be ”naturally” observed in culture, a comparison was performed with a 10‐day slice incubated for 2.5 days at 4°C. As expected, the slice showed a higher, uniform, and fluorescent signal after incubation than previously (Figure 2c) suggesting that the basal cell death rate is produced naturally in culture. Furthermore, this was also contrasted by a study on the hippocampal organotypic slices exposed to glucose and oxygen deprivation. Cultures in hypoxia presented neuronal damage with an high PI signal specifically detected in the cornu Ammonis and the dentate gyrus (Allard, Paci, Vander Elst, & Ris, 2015).

To know if slices could develop any inflammation in culture, microglial activation was investigated after 22 days by immunohistochemistry with an anti‐Iba1 antibody, a marker of early activation. Confocal microscopy revealed that the microgliocytes presented a ramified phenotype with numerous fine branching processes in the control slices. As such, these cells modified their morphology toward a macrophagic phenotype in response to lipopolysaccharide (LPS) treatment, an inflammatory stimulant (Figure 3). It has long been thought that ramified microglia were “resting” in the healthy brain, however, evidence suggests that microglial cells are highly active in their presumed resting state, continually screening their microenvironment with extremely motile processes and protrusions (Nimmerjahn, Kirchhoff, & Helmchen, 2005). Moreover, it has been shown that ramified microglia not only monitor their microenvironment but also protect hippocampal neurons under pathological conditions (Vinet et al., 2012). Thus, according to the present experimental conditions, these observations suggest that hippocampal organotypic slices present active and neuroprotective ramified microglia.

After checking the state of the hippocampal slices in culture, dendritic spines were analyzed 3 weeks after birth to evaluate their synaptic maturity before the electrophysiological investigations. To detect and visualize these small protuberances, F‐actin was targeted by phalloidin revealing numerous spots on the surface of dendrites, probably dendritic spines (Figure 4a). Buchs et al. (1993) showed that the density of synaptic contacts increased significantly during the first 3 weeks in culture in the *stratum radiatum,* reaching a similar level to that observed in 1‐month‐old rats. This study, carried out on 5‐day‐old rats, demonstrated that the number of synapses was very large around 3 weeks in culture, representing an average of 20 synapses per 100 μm^2^ and increased a little to 25 synapses per 100 μm² one week later. The comparison between organotypic slices taken from 5‐day‐old rats, and maintained for 22–30 days in culture, and acute slices from 30‐day‐old rats revealed that synaptic density in culture was slightly lower than *in vivo,* suggesting that the interface method of hippocampal organotypic cultures can allow studies of synaptic development *in vitro*. At the same time, a progressive increase in the amplitude of the maximal synaptic field potentials was measured in the CA1 area of cultured slices (Muller et al., 1993).

In order to carry out quantitative and qualitative analysis of dendritic spines in the present model, organotypic slices were transfected with EGFP‐plasmid. By using the biolistic method, which is an effective and straightforward technique to transfect post‐mitotic neurons deep in tissues (O'Brien & Lummis, 2013), spine populations were studied and characterized from a series of three‐dimensional confocal pictures, representing the total area of each transfected neuron (Figure 4b). Semi‐automatic analysis performed with the Filament tracer software revealed that spine density on apical dendrites represented on average 3.21 spines/10 μm (Figure 5b). These values were lower than in another study carried out on 8 to 9‐day‐old rats showing that after 2 and 3 weeks in culture average spine density was 7 and 11 spines/10 μm respectively (Collin, Miyaguchi, & Segal, 1997). This difference may occurs a result of age of the sacrificed neonate rats, which were older in this work, and also to the analysis method used. Indeed, the selection of dendritic segments across neurons could have introduced a bias by the experimenter, contrary to the present analysis method which takes into account the entire apical dendritic surface of the neurons. Qualitative analysis was carried out on each spine of each transfected neuron and an automatic spine classification was applied with R software on the dataset according to the classification method of Weyer et al. (2014). Respective proportions of different spine categories are reported in a table (Figure 5c) and plotted (Figure 5a) for each neuron demonstrating that mushroom‐type spines represent the biggest group, with around 68% of the spine population, and 20% and 12% of stubby and thin‐type spines respectively. Weyer et al. (2014) showed similar results with 70% of mushroom‐type spines, 20% of stubby‐type spines and 10% of thin‐type spines. It is known that spines with large heads (mushroom‐type) are stable and express numerous AMPA receptors contributing to strong synaptic connections, contrary to spines with small heads which are unstable and immature. The structure‐function relationship of spines suggests that large spines are “memory spines” and small spines are “learning spines” (Kasai, Matsuzaki, Noguchi, Yasumatsu, & Nakahara, 2003). With the present analysis, it can be seen that the neuronal network of hippocampal organotypic slices acquired functional maturity according to the proposed *in vitro* conditions.

After having demonstrated that cultured slices presented abundant mature spines and secondarily that axons and dendrites were co‐located in a same plan with cross‐section recommendations (Figure 6), their electrophysiological properties were investigated.

To measure the electrical responses, organotypic slices had to be moved from the culture system to the recording chamber. However, it is very difficult to extract slices from the culture system without causing damage, given that they stick strongly to the insert membrane. In different studies, instruments or pipettes were used to remove slices from the culture insert, but these procedures are very delicate and exert high mechanical pressure onto the tissues, which can lead to axonal damage and/or immune response. The confetti of the hydrophilic PTFE membrane, placed into the insert before use, allowed the safe recovery of the slices with fine pliers. However, it is important to note that this technique is not adapted to an interface chamber, given that confetti tends to float to the surface of aCSF even when silver weights are used for the stabilization. In this case, the submersion method could have been used, but it does not allow for optimal oxygenation of tissues. After trying these different methods, a new configuration is highly recommended (Figure 1). It allows slices to be kept easily and safely in the interface during electrophysiological investigations. Culture inserts can simply be placed into the recording chamber using a designed Plexiglass adaptor piece, achievable by following the supplied plan, and which can be adjusted to any model of the chamber and/or culture insert. Moreover, this system presents the advantage of maintaining slices in conditions as close to those present in culture while infusing slices with an oxygenated and heated aCSF.

Long‐term potentiation can be induced in many different ways, electrically, according to diverse combinations of stimulation, or chemically, with drug administration. In this study, a standardized comparison of three different methods was carried out. The first consisted of three stimulus trains of 100 pulses at 100 Hz with 10 min inter‐train intervals, as described by Frey and Morris (1997) for acute slices. This method had already been validated in the cultured hippocampal slices for which 75% of the slices showed an LTP lasting more than 1 hr with an averaged amplitude of fEPSP of 143.2 ± 3.2% of the baseline after 1 hr of recording (Shimono, Baudry, Ho, Taketani, & Lynch, 2002). Similar to the aforementioned work, the results here showed 72% of the slices exhibiting an LTP (Figure 7b) lasting 113 min on average with an fEPSP amplitude of 176 ± 11% of the baseline (data not shown).

The second tested method was both chemical and electrical using a perfusion of forskolin (50 μmol/L, 5 min) before the stimulation trains. Forskolin is an activator of adenylyl cyclase that increases transmitter release in CA1 presynaptic neurons (Chavez‐Noriega & Stevens, 1992). However, this effect is reversible and it has been demonstrated that forskolin did not induce a long‐term effect on the fEPSP, contrary to the population spikes where a long‐lasting increase of their amplitude was observed (Kwok‐Tung & Po‐Wu, 1999). As expected, just forskolin did not allow to induce the LTP in our cultured slices, only a transient increase of fEPSP was observed, lasting around 30 min after induction (data not shown). Forskolin perfusion was thus coupled with stimulation trains to try to generate the stable LTP. This was successful as good protocol efficacy was achieved with 73% of the slices displaying a sustained increase of fEPSP (Figure 7b) lasting more than 1 hr and reaching 144 ± 8.6% of the baseline.

Finally, the third method was devised from various studies demonstrating the requirement of dopaminergic afferents for persistent long‐term potentiation in the hippocampus. Indeed, it had previously been validated in acute slices from 5 to 6‐week‐old rats that the late phase of LTP was mediated by a dopaminergic modulatory input coupled with adenylyl cyclase activation (Huang & Kandel, 1995). Moreover, another study provided the evidence *in vivo* that D1, and not D5, dopamine receptors are critical for the LTP, spatial learning, and arc expression in the hippocampus (Granado et al., 2008). It is important to specify that dopaminergic afferent axons are preserved in acute slices, contrary to the organotypic slices where severed fibers degenerate. For this reason, it would seem essential to add a dopaminergic agonist during LTP induction protocol in organotypic cultures. In the same way as forskolin, SKF 83822 hydrobromide, a selective dopamine D_1_‐like receptor agonist, perfused alone in the recording chamber did not induce stable LTP (data not shown). However, the infusion of SKF (10 μmol/L, 15 min) followed by three trains of 100 pulses at 100 Hz with 10‐min inter‐train intervals exhibited an efficacy similar to the previous protocols. Indeed, 69% of the slices presented persistent LTP (Figure 7b) lasting on average 136 minutes, with an increase in fEPSP amplitude of 169 ± 10% and an fEPSP slope of 176 ± 19% in comparison with the baseline (Figure 7c). Nevertheless, the most relevant fact was that most of the slices stimulated with this procedure showed a simultaneous increase of synaptic strength in amplitude and slope, as usually observed in acute slices (Figure 7b–c). Thus, a combination of SKF and trains can be suggested to induce the LTP in hippocampal organotypic slices.

Note that different LTP profiles have been encountered according to the investigated cultured slices as well as population spike potentiation (Figure 7b). However, the decision was made not to study the latter because their parameters are often unmanageable by the complexity of the responses they generate.

In conclusion, these experiments on slice viability and microglial expression have validated the used culture protocol that has been slightly modified from the original protocols (Muller et al., 2001; Stoppini et al., 1991) given that slices are viable for several weeks with the structural characteristics of the hippocampus. Moreover, the studies on dendritic spines have shown that neuronal maturity was acquired in culture with a big proportion of mushroom spines. Finally, the most relevant point of this study concerns the description of a new recording configuration specifically adapted to cultured slices. This system allows experimenters to carry out electrophysiological studies on hippocampal organotypic slices while preserving their integrity, which is essential for successful experiments.

## Conflict of Interests

None declared.
